# Gate Enhancing Charge‐Spin Conversion in Organic Chiral Field Effect Transistors

**DOI:** 10.1002/advs.202524175

**Published:** 2026-02-08

**Authors:** Shilin Li, Renjie Hu, Xiangping Zhao, Xi Wang, Wei Qin

**Affiliations:** ^1^ School of Physics State Key Laboratory of Crystal Materials Shandong University Jinan China

**Keywords:** ferromagnetic–ferroelectric heterostructure, interfacial interaction, magnetoelectric coupling, organic ferroelectric field‐effect transistors

## Abstract

Organic ferroelectric field‐effect transistors (OFeFETs) are promising candidates for next‐generation wearable electronics and non‐volatile memory technologies owing to their bistable switching, low power consumption, and mechanical flexibility. Here, room‐temperature organic chiral multiferroic FETs are demonstrated, in which both gate field and chiral field dependence of charge‐spin conversion are studied. Remarkably, the chiral FET presents tens of micrometer chiral signal transport. This long‐range chiral transport provides an ideal platform for probing the interaction between charge and chirality‐induced polarized spin. The interfacial dipoles in the ferroelectric layer could impact the degree of charge carrier localization to further modulate spin polarization, presenting a charge‐spin conversion‐dependent magnetoelectric coupling. Conversely, polarized spin in the organic chiral layer could modify saturated ferroelectric polarization and ferroelectric hysteresis loop, apparently. In addition, when an external magnetic field is applied parallel (antiparallel) to the chiral axis, the OFET shows an enhanced (weakened) chiral magneto‐chiral current, which can also be modulated by remanent polarization.

## Graphical abstract

Room‐temperature organic chiral multiferroic field effect transistors are demonstrated, enabling bidirectional control between spin and charge. Specifically, spin polarization affects the ferroelectric polarization, and ferroelectric polarization, in turn, can modulate spin polarization and spin‐dependent transport. Moreover, tens of micrometer chiral signal transport has been obtained, offering a practical long‐range platform to probe chirality‐induced spin polarization.

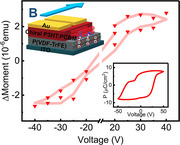

## 1 Introduction

Constructing heterojunctions with ferromagnetic materials and ferroelectric layers to achieve synergistic control of spin and charge within a single device is a widely explored topic in contemporary spintronics research [1, 2, 3, 4, 5]. In such ferromagnetic/ferroelectric (FM/FE) heterostructures, charge‐spin conversion arises from various mechanisms, including electrostatic interactions mediated by interfacial charge modulation [6, 7, 8], strain effects induced by the coupling between magnetostriction and piezoelectricity [9, 10, 11, 12, 13], and interfacial exchange interactions [14, 15]. Reversal of the ferroelectric polarization state can lead to interfacial charge redistribution and the generation of piezoelectric strain, which in turn regulates the magnetic anisotropy, coercivity, and spin configuration of the adjoining ferromagnetic layer [16, 17, 18]. Additionally, the magnetic ordering within the ferromagnetic layer can reciprocally influence ferroelectric polarization behavior through the converse magnetoelectric effect [19, 20]. Beyond inorganic systems, organic flexible composites have also made significant strides [21], with promising applications in biomedicine [22, 23], energy conversion [24], and wearable electronics [25, 26].

In conventional organic field‐effect transistors (OFETs), modulation of carrier concentration via gate voltage is a fundamental phenomenon [27]. Beyond that, applying a gate voltage can also prompt charge transfer [28], affect the mobility of charge carriers [29, 30], and improve injection efficiency at the source and drain electrodes [31]. The strong gate electric field can further influence molecular dipole interactions, thereby altering molecular packing [32] and luminescence [33, 34] in OFETs. OFeFETs, which employ organic ferroelectric materials as gate dielectrics [35, 36, 37, 38, 39], have evolved from traditional memory devices into versatile platforms for applications [38]. Their transport characteristics are governed by carriers trapping [36] and interfacial polarization switching [35]. Multiferroic FETs, which combine ferroelectric and ferromagnetic order, are emerging as candidates for next‐generation electronics with non‐volatile charge‐spin conversion. Electrically gated spin‐to‐charge conversion in inorganic heterostructures generates an interfacial spin density coupled to the ferroelectric polarization, which have considerable significance for all‐electrical spin generation [40, 41]. Meanwhile, gate generation, manipulation, and detection of spin polarization in inorganic single‐crystalline tellurium nanowires are demonstrated, which opens the path to exploit the design of inorganic solid‐state spintronic devices [42]. Despite advances in spin control within inorganic systems, modulating spin properties effectively via gate electrodes in organic FETs remain challenging. Advancing OFeFETs based on organic FM/FE heterojunctions requires a deeper understanding of the gate dependence of charge‐spin conversion ability and how the charge‐spin conversion depends on the chiral field.

In this work, organic chiral multiferroic FETs were constructed to investigate both gate field and chiral field dependence of charge‐spin conversion. The introduction of the organic chiral magnetic layer leads to marked changes in the saturated ferroelectric polarization and ferroelectric hysteresis, providing direct evidence of interfacial charge‐spin coupling. By varying the gate voltage to control the ferroelectric polarization strength, the spin properties in the ferromagnetic layer show a clear response, where non‐volatile spin polarization can be efficiently gated. Furthermore, the chiral OFET shows an enhanced (weakened) magneto‐chiral current when an external magnetic field parallel (antiparallel) to the chiral axis is applied. Overall, coherent control of spin polarization and chirality‐dependent transport through ferroelectric polarization in chiral organic FM/FE FETs is demonstrated.

## 2 Results and Discussion

**FIGURE 1:**
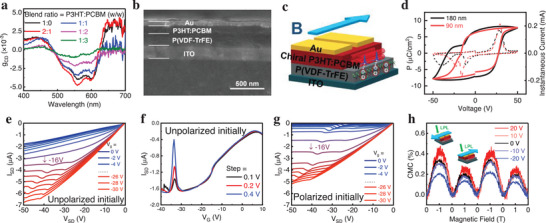
Structure and Transport Properties of OFET Based on FM/FE Heterostructures. (a) Circular dichroism dissymmetry factor (*g*_CD_) of chiral P3HT films with different PCBM ratios. (b) Cross‐sectional SEM image of the OFET device; the inset shows CD spectrum of the P3HT:PCBM layer. (c) Illustration of the OFET architecture and the orientation of the externally applied magnetic field during testing. (d) Ferroelectric loops and corresponding instantaneous currents measured for devices with different P3HT:PCBM thicknesses and a constant P(VDF‐TrFE) thickness (device structure: Ag/P3HT:PCBM/P(VDF‐TrFE)/ITO). (e) Output characteristics of the OFET device without any pre‐applied gate voltage. (f) Transfer characteristics of the OFET device measured at different sweep rates without any pre‐applied gate voltage. (g) Output characteristics after a pre‐applied gate voltage of +40 V. (h) CMC at 300 K under 532 nm linear polarized light (LPL) illumination, and the insets show the direction of the applied magnetic field (light blue arrow) and polarization states of light. Here, the CMC is defined as CMC = $\frac{I(B)-I(0)}{I(0)}$, The voltage refers to the gate voltage applied before the test, while the gate was held at 0 V during data acquisition.

Ferroelectric material Poly(vinylidene fluoride‐co‐trifluoroethylene) [P(VDF‐TrFE)] is used owing to its excellent piezoelectric properties and environmental stability. With a high dielectric constant, it is frequently employed as the dielectric material in low‐voltage OFETs [35, 36, 43]. Poly(3‐hexylthiophene‐2,5‐diyl) (P3HT) is a highly crystalline polymer known for its excellent charge carrier mobility and film‐forming properties, making it one of the most widely used *p*‐type conducting polymers for OFET. Here, chiral P3HT nanostructures are induced to form a helical supramolecular structure with a pronounced circular dichroism (CD) signal (Figure 1a). Upon blending with Phenyl‐C_61_‐butyric acid methyl ester (PCBM), charge transfer occurs between P3HT and PCBM, and this charge transfer, along with the induced structural order, gives rise to spontaneous spin polarization (Figure S1) [44, 45, 46]. Structurally, a distinct peak at 2*θ* = 5.37° is observed in chiral P3HT films (Figure S2), which is assigned to P3HT (100) lamellar stacking and indicates an a‐axis oriented microcrystalline packing [47]. Notably, increasing the PCBM content enhances phase separation; while it does not eliminate the ordered lamellar stacking, it progressively disrupts the chiral morphology, ultimately rendering the nanofibers indistinguishable (Figure S3). Consistent with this structural disruption, the CD dissymmetry factor decreases rapidly with increasing PCBM ratio (Figure 1a). Here, the heterojunction consisting of P(VDF‐TrFE) and P3HT:PCBM at a 1:1 mass ratio, which is selected as a representative composition balancing chirality, charge transfer, and film formation, is fabricated, with a cross‐sectional scanning electron microscopy (SEM) image and device schematic shown in Figure 1b,c, respectively. Atomic force microscope (AFM) measurements indicate that the P(VDF‐TrFE) dielectric surface is relatively smooth, with a root‐mean‐square (RMS) roughness of 0.94 nm (Figure S4), falling into the sub‐nanometer regime that is commonly considered optimal for organic electronics [48]. The ferroelectric hysteresis loop of the heterojunction structure deviates significantly from that of the intrinsic ferroelectric layer (Figures 1d; S5–S8). As shown in Figure 1d, a distortion appears on the left side of the loop, indicative of interfacial coupling between the two functional layers. Notably, this left‐branch distortion is still present when the chiral semiconductor is replaced by the achiral control (Figure S9), indicating that it is induced by the ferromagnetic–ferroelectric interfacial interaction and is therefore not dependent on whether P3HT is chiral.

Based on the interfacial coupling, output characteristics of the OFET device present a pronounced response (Figure 1e,f). In addition, a pre‐polarization voltage leads to the polarization of P(VDF‐TrFE), where the characteristics of the OFET become strongly related to the interfacial coupling (Figures 1g; S11 and S12). Accordingly, the field effect mobility (*µ*), on/off current ratio ($\frac{I_{\mathrm{on}}}{I_{\mathrm{off}}}$), and threshold voltage (*V*_th_) under different gate pre‐bias conditions are extracted from transfer characteristics (Figure S13) and summarized in Table S1. Also, by applying an external magnetic field parallel (antiparallel) to the chiral axis, the OFET shows an enhanced (weakened) chiral‐magneto‐conductance (CMC) (Figure 1h). Furthermore, owing to the interfacial interaction, the magnitude of the CMC could be modulated by the remanent polarization of the ferroelectric layer. In the following, more discussion and the mechanism behind the phenomena will be further studied to understand the interfacial charge‐spin conversion.

**FIGURE 2:**
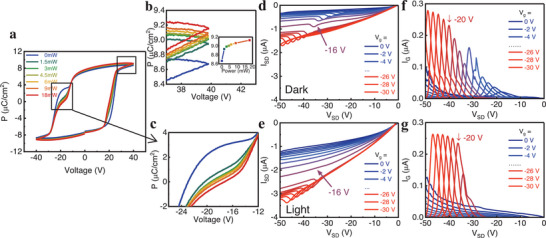
Light‐Induced Modulation of Polarization and Transport. (a) Ferroelectric loops of the P3HT:PCBM/P(VDF‐TrFE) heterojunction under different light intensities. (b) and (c) are zoomed‐in views of selected portions of (a), providing more details, the inset in (b) shows the light‐intensity dependence of the maximum polarization. (d) and (e) show the output characteristics of the OFET in the dark and under 532 nm laser illumination, respectively. The corresponding gate currents are shown in (f) and (g). A gate voltage of +20 V was applied prior to the measurements of (d–g).

The P3HT:PCBM layer could modulate the external dipolar alignment of the P(VDF‐TrFE), thereby affecting its ferroelectric properties (Figures 1d and 2a–c). Under illumination, this modulation becomes more pronounced, with both the maximum and remanent polarization increasing (Figure 2b). Laser exposure generates more charge carriers in P3HT:PCBM, enhancing its built‐in electric field to further reduce electron localization. As a result, weakly‐localized charges at the interface will exhibit good itinerant properties to weaken the interfacial coupling, allowing the P(VDF‐TrFE) layer to polarize more readily under an external electric field, resulting in an increase in both maximum and remanent polarization. Higher carrier concentration also delays the reduction of the transient current to zero, resulting in a less distorted current profile under illumination (Figure 2c). In addition, it should be noted that light illumination could introduce plenty of electron‐hole pairs in P3HT:PCBM, which should increase the number of dipoles and thereby strengthen the dipole–dipole interactions at the interface. However, the result shows that the interfacial interaction is weakened, which suggests that the interfacial dipole–dipole coupling in this heterojunction is inherently weak and can be ignored, thereby facilitating the observation and modulation of charge‐spin conversion.

Carrier transport in the P3HT:PCBM layer is suppressed via significant interfacial interaction induced by the remanent polarization of a pre‐polarized P(VDF‐TrFE) layer, resulting in a reduced source‐drain current (*I*_SD_) compared to the unpolarized condition (Figures 1e,g; S11–S15). As shown in Figure 1g, the *I*_SD_ is consistently lower regardless of the applied gate voltage (*V*_G_). Notably, when *V*_G_ ranges from 0 to −16 V, the difference in current between the two cases is relatively large. However, when *V_G_* exceeds –16 V, the current profiles tend to become similar in shape, differing mainly in magnitude. This behavior indicates that when *V*_DG_ = *V*_D_ −*V*_G_ = +16 V, corresponding to *V*_G_ = −16 V, heterojunction returns to a state nearly equivalent to the unpolarized case due to the depolarization. Nevertheless, the depolarization induced here is not completely equivalent to an initial unpolarized state, since the voltage is applied only on one side of the channel, which means the depolarization occurs only on one side of the device. As a result, the *I*_SD_ remains different in magnitude compared with Figure 1e.

As shown in Figure 1e–g, it is noted that some anomalous current peaks were observed in the output and transfer characteristics, which can be attributed to the polarization switching of P(VDF‐TrFE). Once the gate voltage exceeds the ferroelectric material's coercive field, the polarizing process of P(VDF‐TrFE) induces transient currents, which can interfere with the *I_SD_*, resulting in anomalous current peaks. To rule out an injection‐barrier‐modulation origin, we measured both the output and transfer characteristics at different sweep rates (Figures 1f; S16). The peak amplitude increases as the sweep rate becomes faster, which is consistent with ferroelectric switching behavior because the transient current scales with the polarization switching rate, and it is opposite to the trend expected for barrier modulation or charge trapping, which typically become more pronounced at slower sweeps. In the unpolarized case (Figure 1e), weaker interfacial coupling makes these polarization‐induced currents less noticeable within the 0 to −16 V gate range. Similarly, under illumination, the reduced interfacial localization caused by increasing built‐in field weakens the interfacial coupling, making polarization‐induced current variations harder to detect in *I*_SD_ (Figures 2d–g; S19). Lowering the temperature to 100 K enhances interfacial interactions, making the transient current observable even without pre‐bias or under illumination (Figures S19 and S20), despite incomplete ferroelectric polarization at low temperatures (Figures S6 and S7). This indicates that temperature‐induced interfacial enhancement is more significant. Furthermore, illumination reduces the gate‐source voltage required to trigger polarization in P(VDF‐TrFE), consequently lowering the source‐drain voltage threshold for the anomalous current peak (Figures 2f,g; S17b), which is consistent with the narrower ferroelectric loop under illumination (Figure 2a).

**FIGURE 3:**
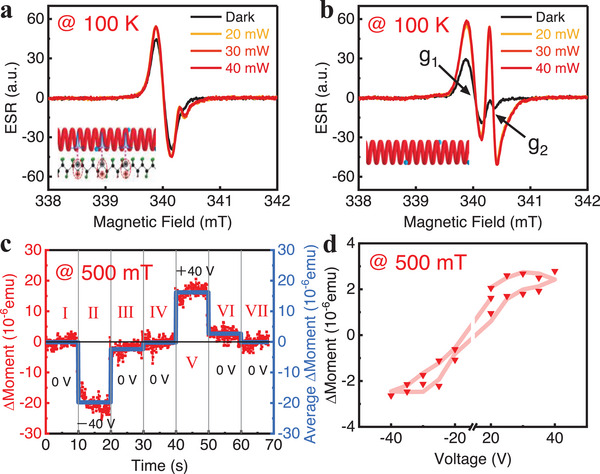
Control of Spin Polarization. ESR signals of P3HT:PCBM/P(VDF‐TrFE) heterojunction (a) and pristine P3HT:PCBM (b) under dark and 532 nm laser illumination at different power levels. The ESR spectrum in (b) shows two g‐factors: g_1_ for P3HT holes and *g*_2_ for PCBM electrons. (c) Time‐resolved magnetization at 500 mT showing responses to different electric polarization stages (each 10 s): I. initial, II. polarization, III. remanence, IV. depolarization, V. reverse polarization, VI. reverse remanence, and VII. full depolarization. (d) Magnetization as a function of remanent polarization under different polarized voltages, all data were collected at zero applied voltage and a 500 mT magnetic field. The red inverted triangles depict the experimental data, and the pink curve is a Savitzky–Golay smoothing presented for visual clarity. All data shown in (c) and (d) were from Ag/P3HT:PCBM/P(VDF‐TrFE)/ITO devices.

The spin polarization in P3HT:PCBM can be effectively modulated by the ferroelectric layer. Due to strong electron localization at the interface induced by the interfacial coupling, a weaker electron spin resonance (ESR) signal from PCBM electrons is observed in the P3HT:PCBM/P(VDF‐TrFE) heterojunction (Figures 3a; S24), compared to the pristine P3HT:PCBM blend (Figure 3b). Moreover, the spin‐spin relaxation time *T*_2_ and spin‐lattice relaxation time *T*_1_ are estimated by fitting power‐dependent CW‐ESR data (Figure S25), yielding *T_1_* ≈536 ns and *T_2_* ≈30.8, which indicates a characteristic spin‐state lifetime on the order of hundreds of nanoseconds. The origin of P3HT:PCBM charge transfer crystals ferromagnetism has been studied in our previous works [46, 49]. It is known that stronger localization of electrons will result in a larger spin polarization. By gate‐polarizing the P(VDF‐TrFE) ferroelectric layer, aligned dipoles will effectively lead to the localization of electrons, generating stronger spin polarization. As shown in Figure 3c, a gate can reversibly tune this spin polarization. Applying a negative gate polarizes P(VDF‐TrFE) (stage II in Figure 3c), and the magnetization of P3HT:PCBM remains in a modified state even after gate removal (stage III in Figure 3c), recovering only upon depolarization (stage IV in Figure 3c). Conversely, a positive gate enhances spin polarization (stage V–VI in Figure 3c). Here, applying a 40 V bias induces a magnetization change of ΔM ≈20 × 10^−6^ emu. Using $\alpha =\frac{\mu_{0\Delta M}}{\Delta E}$, the magnetoelectric coupling coefficient is calculated to be 8.1 × 10^3^ mV/(cm·Oe). By sweeping the gate magnitude and polarity and tracking the resultant magnetization changes, a clear magnetization‐voltage hysteresis loop can be obtained (Figure 3d). These results demonstrate that the gate dependence of ferroelectric polarization of P(VDF‐TrFE) can directly modulate the spin polarization in P3HT:PCBM through interfacial coupling, illustrating a reversible conversion between charge and spin. The hysteretic relationship confirms that the ferroelectric domains can set the spin polarization state in the organic semiconductor layer without the need for a continuous power supply. Furthermore, the robustness of this magnetoelectric coupling at the interface suggests potential for developing novel memory elements, where information can be written electrically and read magnetically.

**FIGURE 4:**
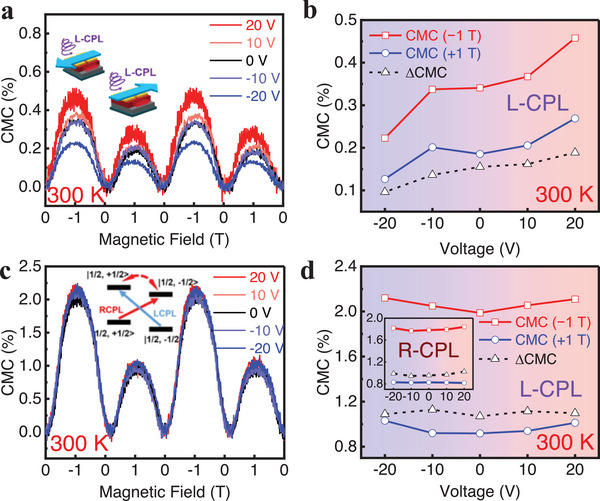
CMC under Circularly Polarized Excitation. (a) CMC of the OFET at 300 K under L‐CPL, and the insets show the direction of the applied magnetic field (light blue arrow) and the polarization states of light. (b) Voltage dependence of CMC under L‐CPL at 300 K, and Δ(CMC) is defined as: Δ(CMC) = CMC(−1 T) − CMC(+1 T). (c) CMC of the OFET at 100 K under L‐CPL. The inset shows the schematic of photoexcited electron relaxation in chiral P3HT:PCBM charge‐transfer complexes. (d) Voltage dependence of CMC under L‐CPL at 100 K, and the inset shows data under R‐CPL. In all measurements, the gate was pre‐biased before testing and held at 0 V during data acquisition.

Besides the tunability of spin polarization by gate voltage, circularly polarized light and magnetic field could also effectively modulate the chirality dependence of spin polarization. As shown in Figure 4a,c, the application of an external magnetic field results in an increased *I*_SD_. This phenomenon can be attributed to the enhanced spin polarization, which will cause the spin state to be more localized. The more localized electronic state caused by the magnetic field will reduce the attraction to holes, thereby enhancing the transport of holes in chiral P3HT nanowires to further increase *I*_SD_. Interestingly, the modulation of the channel current by the magnetic field is highly dependent on the direction of the field. As shown in Figure 4a, when switching magnetic field from parallel to antiparallel to the axial direction of the chiral P3HT nanowire, the conductance presents different magnitudes. The helical arrangement of P3HT chains along the transport direction introduces a chiral magnetic field *B*_chiral_, making the effective magnetic field *B*_effective_ = *B*_external_ + *B*_chiral_, which breaks symmetry between positive and negative magnetic fields. Therefore, a parallel alignment enhances the effective field, leading to more efficient current control compared to the antiparallel case (Figure 4a,c). This CMC phenomenon is not observed in the achiral devices (Figure S29), confirming that the CMC originates from the chiral architecture. Moreover, chirality‐associated transport is highly sensitive to the chiral morphology: as the PCBM ratio increases, the CMC signal gradually weakens and can even vanish (Figure S30), consistent with enhanced phase separation and disruption of the chiral structures, which further undermines chirality‐related spin transport. To quantify the magnetic‐field asymmetry in the CMC response, we define *g*_CMC_ = $\frac{\mathrm{CMC}(-1\;T)\;-\;\mathrm{CMC}(+1\;T)}{\mathrm{CMC}(-1\;T)+\;\mathrm{CMC}(+1\;T)}$, and use *g*_CMC_ as a practical metric to assess whether chirality‐related transport remains distinguishable. A small *g*_CMC_ value close to zero, as typically observed in achiral OFETs, indicates the absence of chirality‐related signal transmission. In contrast, for chiral OFETs, the FET channel used here is 40 µm, which means that chirality‐dependent signal transmission can exceed this value, which is a very important distance for the device design in the future.

Due to the interfacial charge–spin interaction, the spin‐dependent transport should be gate‐tunable. Specifically, a positive gate bias enhances the CMC, while a negative gate bias suppresses it (Figure 4a,b). Under positive bias, the dipole orientation in the ferroelectric layer deviates from that in the P3HT:PCBM blend, resulting in more PCBM electrons bound at the interface. Therefore, the magnetic field will regulate the localization of electrons more effectively, reducing the attraction to holes and enhance the transport of holes to increase the CMC. For an applied negative gate, the polarized dipole at the interface will reduce the concentration of bound PCBM electrons at the interface, where a smaller CMC is observed. Moreover, a decrease in temperature suppresses the polarization of the ferroelectric layer (Figures S6 and S7). Although interfacial interactions are strengthened at lower temperatures, the applied voltage becomes insufficient to effectively modulate the ferroelectric polarization, resulting in diminished gate tunability of the CMC (Figure 4c,d). It is also noteworthy that the chirality‐dependent transport is sensitive to the handedness of incident circularly polarized light (CPL). Under left‐handed CPL (L‐CPL), the CMC response is more pronounced than under right‐handed CPL (R‐CPL) (Figure 4d). This is because the electron‐hole pairs excited by L‐CPL have a smaller binding energy (inset of Figure 4c), allowing the magnetic field to more effectively increase the triplet states population and enhance dissociation thus yielding a stronger CMC (Figure 4d).

## 3 Conclusion

In summary, OFETs incorporating FM/FE heterojunctions are constructed using the highly crystalline, chiral ferromagnetic charge‐transfer complex P3HT:PCBM and the ferroelectric material P(VDF‐TrFE), where strong interface coupling was observed. The saturated ferroelectric polarization, ferroelectric loop, and ESR signals show significant differences after the introduction of the organic chiral magnetic layer. The orientation of the dipoles in P(VDF‐TrFE) influences the spin polarization and localization in P3HT:PCBM and a voltage‐magnetic moment hysteresis loop can be observed. The ferroelectric polarization of P(VDF‐TrFE) can directly modulate the spin polarization in P3HT:PCBM through interfacial coupling to present a conversion between charge and spin. By increasing the gate to adjust the polarization strength of the ferroelectric layer, the spin properties in the magnetic layer show a significant response. In addition, by applying an external magnetic field parallel (antiparallel) to the chiral axis, the OFET shows an enhanced (weakened) CMC.

## 4 Experimental Section

### 4.1 Materials

Thirty milligrams of P3HT was dissolved in 1 mL of o‐dichlorobenzene and stirred at 45°C for 2 h to ensure complete dissolution, followed by natural cooling to room temperature. Subsequently, 2 mL of R‐(+)‐limonene was added into the solution, and the mixture was stirred continuously for 24 h to promote chiral induction. Afterward, 0.1 mL of acetonitrile was added, and the mixture was stirred for another 2 h and then left to stand for 48 h to obtain the chiral P3HT solution. For the achiral P3HT solution, the R‐(+)‐limonene was replaced with o‐dichlorobenzene, and all other steps were identical to those used for preparing the chiral solution. For the preparation of the donor–acceptor blend, 10 mg of PCBM was dissolved in 1 mL of the chiral/achiral P3HT solution and stirred for 2 h, producing a 1:1 molar ratio chiral/achiral P3HT:PCBM blend solution. For other P3HT:PCBM mass ratios, the corresponding amount of PCBM was weighed and mixed with the chiral P3HT solution to obtain the desired blends. In parallel, 30 mg of P(VDF‐TrFE) was dissolved in 1 mL of tetrahydrofuran (THF) with continuous stirring for 12 h to form the ferroelectric solution. P3HT, P(VDF‐TrFE), and R‐(+)‐limonene were obtained from Sigma‐Aldrich, PCBM from 1‐Material.

### 4.2 Device Fabrication

ITO substrates were cleaned sequentially in an ultrasonic bath with deionized water, ethanol, acetone, and isopropanol (20 min each), followed by nitrogen drying. The substrates were then treated under UV irradiation for 20 min to remove residual organic contaminants and enhance surface energy. In a nitrogen‐filled glovebox, 30 µL of the P(VDF‐TrFE) solution was deposited onto ITO substrates by spin‐coating at 9000 rpm, followed by annealing at 135°C for 10 min. This step facilitated the removal of THF solvent and promoted crystallization of the ferroelectric β‐phase. Once cooled to room temperature, 10 µL of the chiral P3HT:PCBM blend solution was spin‐coated onto the P(VDF‐TrFE) dielectric layer at 3000 rpm. Notably, the o‐dichlorobenzene/limonene mixture did not damage the ferroelectric underlayer. The films were then annealed at 100°C for 10 min to remove the solvents. Thus, the chiral P3HT:PCBM/P(VDF‐TrFE) multiferroic heterojunction was obtained. For electrode deposition, thermal evaporation was carried out under high vacuum. Depending on the characterization requirements, either 100 nm Ag or 50 nm Au was selected. Ag electrodes were used for ferroelectric and magnetic measurements (device configuration: ITO/P(VDF‐TrFE)/chiral P3HT:PCBM/Ag), while Au electrodes were adopted for OFET characterization (ITO/P(VDF‐TrFE)/chiral P3HT:PCBM/Au), in which the P(VDF‐TrFE) layer functioned as the dielectric and the Au channel length was 40 µm.

### 4.3 Characterization

The ferroelectric *P–V* hysteresis loops were measured using a Radiant Precision LC tester. Magnetization–magnetic field (*M–H*) loops were characterized using a vibrating sample magnetometer (VSM, Lakeshore 8604), with voltage supplied by a Keithley 2400. Electrical characteristics including output curves were obtained using a Keithley 4200A‐SCS in a probe station. CD spectra were measured using a JASCO J‐810 spectrometer. Morphological characterization was carried out using scanning electron microscopy (SEM, Hitachi SU8000, Bruker Nano GmbH, Germany). The thickness, morphology, and phase images of the chiral P3HT:PCBM layer and the morphology of P(VDF‐TrFE) films were measured by SHIMADZU SPM‐9700HT atomic force microscope (AFM). For thickness measurement, reference samples consisting of a bare P(VDF‐TrFE) layer and heterojunctions with different chiral P3HT:PCBM thicknesses were prepared. By subtracting the thickness of the pure P(VDF‐TrFE) layer, the effective thickness of the chiral P3HT:PCBM layer was obtained. XRD measurements were performed on a Rigaku SmartLab 3 kW diffractometer using a Cu‐target X‐ray tube. ESR spectra were obtained on an EPR200‐Plus instrument working at X‐Band, CIQTEK Co. Ltd. The 532 nm laser used in the experiments was an MGL‐U‐532‐300 mW from Changchun New Industries Optoelectronics Technology Co., Ltd. Circularly polarized light was modulated based on a GL5‐A Glan‐Laser polarizer and a zero‐order quarter‐wave plate (WPQ05M‐532) from Thorlabs.

## Author Contributions

The idea of the work is generated from W.Q.; experiments are designed by S.L. The manuscript was written through contributions of all authors. All authors have given approval to the final version of the manuscript.

## Funding

This work was supported by the Key R&D Program of Shandong Province, China (2025CXPT204); the Major Program of Shandong Province Natural Science Foundation (ZR2024ZD45); the NSFC (92361301); the Shandong Basic Research Zone (2025SFRZ03).

## Conflicts of Interest

The authors declare no conflicts of interest.

## Supporting information




**Supporting File**: advs74324‐sup‐0001‐SuppMat.docx.

## Data Availability

The data that support the findings of this study are available in the supplementary material of this article.
