# Supplementary material for: Gate Enhancing Charge‐Spin Conversion in Organic Chiral Field Effect Transistors
Source: Adv Sci (Weinh). 2026 Feb 8;13(22):e24175. doi: 10.1002/advs.202524175 (PMC13088329; doi:10.1002/advs.202524175)
Supplement: Supplementary file 1 — Supporting File: advs74324‐sup‐0001‐SuppMat.docx. [file ADVS-13-e24175-s001.docx]

Supporting Information

Gate Enhancing Charge-spin Conversion in Organic Chiral Field Effect Transistors

Shilin Li, Renjie Hu, Xiangping Zhao, Xi Wang, and Wei Qin*

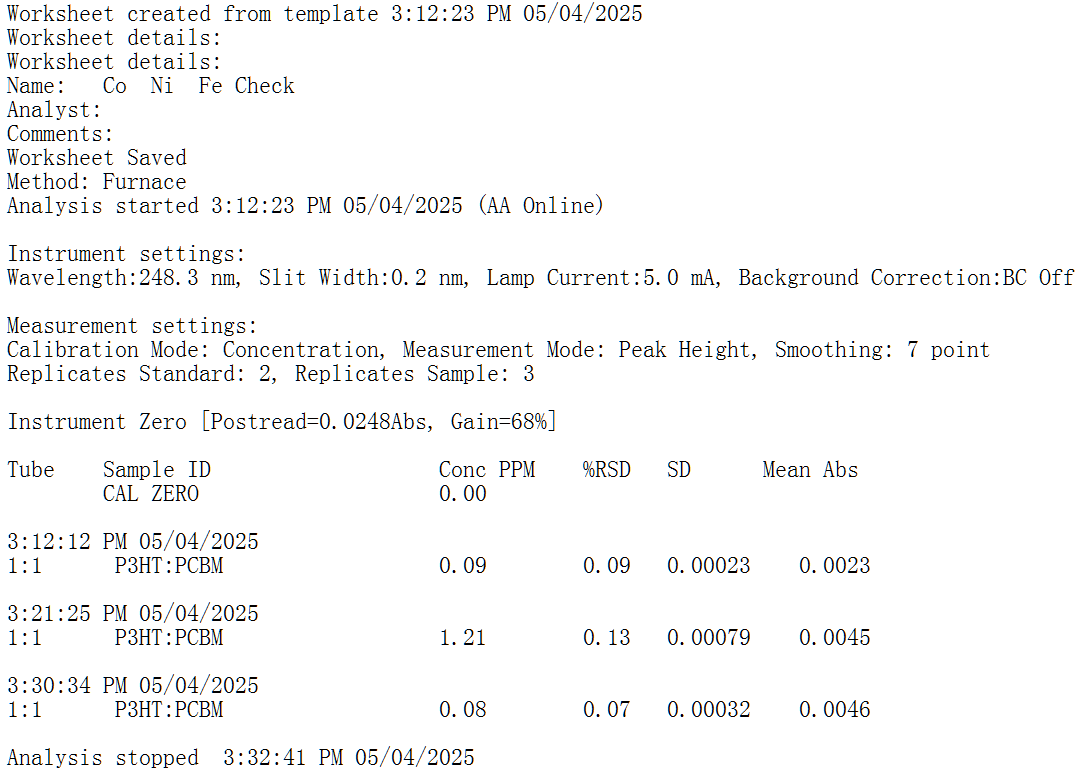


**Figure. S1. *M–H* loop of chiral P3HT:PCBM and Fe, Co, Ni concentrations analysis.**

**Figure S2. XRD characterization.** (a) XRD pattern of P(VDF-TrFE). (b) XRD patterns of chiral P3HT films with different PCBM ratios, including the P(VDF-TrFE) layer.

The peak at 2θ = 5.37° corresponds to the P3HT (100) lamellar stacking and indicates an a-axis oriented microcrystalline packing, where the polymer backbone tends to be parallel to the substrate and the alkyl side chains tend to be perpendicular to the substrate. Notably, this diffraction peak remains present across all PCBM ratios, indicating that the introduction of PCBM does not completely destroy the ordered lamellar packing of P3HT.


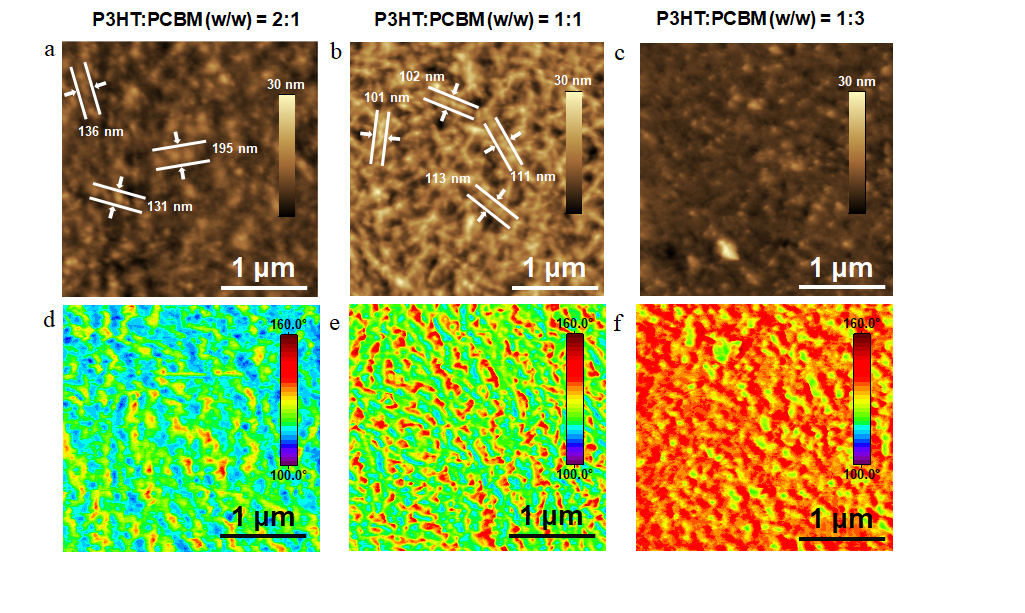


**Figure S3.** **AFM height and phase images of P3HT:PCBM films at different blend ratios.** AFM height images for P3HT:PCBM mass ratio of 2:1 (a), 1:1 (b) and 1:3 (c). AFM phase images for P3HT:PCBM mass ratio of 2:1 (d), 1:1 (e) and 1:3 (f).

The incorporation of PCBM significantly weakens the chiral P3HT nanostructure. As shown in the AFM height images (Figure S3a-c), the helical morphology is largely preserved at low PCBM loading but becomes progressively disrupted as the PCBM content increases. Quantitatively, the average fiber diameter decreases from ~154 nm at a P3HT:PCBM mass ratio of 2:1 to ~106 nm at 1:1, indicating gradual thinning and fragmentation of the helical network. At a 1:3 mass ratio, the chiral nanofibers become indistinguishable.

To assess PCBM-dependent phase separation, AFM phase images were further provided (Figure S3d–f). Because P3HT and PCBM differ in mechanical properties such as stiffness and adhesion, distinct domains yield different phase responses. With increasing PCBM ratio, the phase contrast becomes more pronounced, indicating enhanced phase separation. At higher PCBM content, PCBM increasingly covers the surface, and the phase response becomes more uniform. These observations confirm that PCBM strongly modulates the phase morphology and the degree of phase separation in P3HT:PCBM films.

**Figure S4. Effect of dielectric surface roughness on charge-spin conversion and CMC.** AFM height images of films with different surface roughness values: (a) RMS roughness = 0.94 nm and (c) 1.52 nm (annealing at 150 °C). White lines mark the line-scan positions, and the corresponding height profiles are shown in (b) and (d). (e) Ferroelectric hysteresis loops of P(VDF-TrFE) heterojunctions with different surface roughness values. (f) CMC of the heterojunction with an RMS roughness of 1.52 nm under 532 nm LPL. The voltage refers to the gate voltage applied before the test, while the gate was held at 0 V during data acquisition.

Increasing roughness has a modest influence on both the ferroelectric properties and the charge-spin conversion behavior. The ferroelectric hysteresis loops exhibit slightly reduced remanent and maximum polarization for the higher-roughness sample (Figure S4e). This reduction can be attributed to the increased surface roughness, which lowers the effective polarizability of the ferroelectric layer, or to a decreased β-phase content in P(VDF-TrFE) that weakens its ferroelectric performance. In addition, the distortion of the ferroelectric hysteresis loop also changed, which reflects a change in interfacial interactions rather than the β-phase content. Specifically, the increased roughness modifies the local coupling between the ferromagnetic and ferroelectric layers, thereby altering their interfacial interaction.

Compared with Figure 1h, Figure S4f shows lower asymmetry between positive and negative magnetoconductance and enhanced CMC modulation by ferroelectric polarization. Higher roughness alters the interfacial environment in two coupled ways. On the one hand, it makes the local electric field at the P3HT:PCBM/P(VDF-TrFE) interface more heterogeneous, increasing the effectiveness of ferroelectric polarization in modulating spin-related transport. On the other hand, it disrupts the ordering of chiral P3HT fibrils in the channel, weakening the effect of B_chiral_, which directly suppresses the asymmetry between positive and negative magnetoconductance.


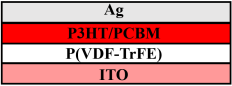


**Figure S5. Ferroelectric loops of the P3HT:PCBM/P(VDF-TrFE) heterojunction.** Ferroelectric loops of the P3HT:PCBM/P(VDF-TrFE) heterojunction, the inset shows the device structure and the driving voltage was applied from the ITO electrode during the test.

Compared with the test which driving voltage was applied from the Ag electrode (Figure 1c), the distortion appears on the other side of the loop.


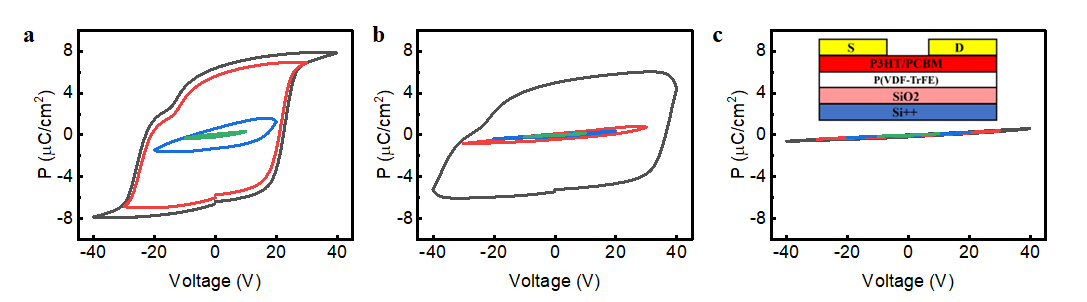


**Figure S6.** **Ferroelectric loops of the P3HT:PCBM/P(VDF-TrFE) heterojunction in the OFET device.** Ferroelectric loops of the P3HT:PCBM/P(VDF-TrFE) heterojunction at 300 K (a), 200 K (b) and 100 K (c) in the OFET device, and the inset of (c) shows the device structure.

In the OFET device, the full ferroelectric loop can be observed, suggesting the ferroelectric layer is capable of full polarization driven by the gate voltage and the source or drain voltage. Moreover, at low temperatures, the ferroelectric layer in the heterojunction cannot achieve full polarization under the same applied voltage. Moreover, increasing applied voltage, a complete polarization curve still cannot be obtained until the device is damaged.


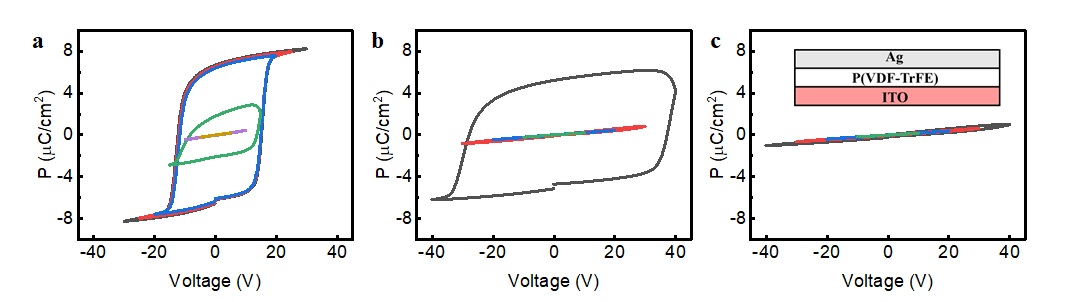


**Figure S7. Ferroelectric loops of bare P(VDF-TrFE).** Ferroelectric loops of bare P(VDF-TrFE) at 300 K (a), 200 K (b) and 100 K (c), and the inset of (c) shows the device structure. A complete polarization curve still cannot be obtained at low temperature.


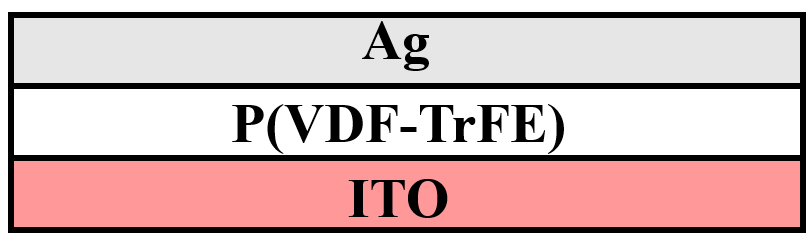


**Figure S8. Ferroelectric loops of bare P(VDF-TrFE) under dark and 532 nm laser illumination conditions, and no loop distortion was observed.**

**Figure S9. Ferroelectric hysteresis loop of the achiral P3HT:PCBM/P(VDF-TrFE) heterojunction.**

**Figure S10. Output characteristics of the OFET device based on chiral P3HT:PCBM at 300 K (a), 100 K (b), 50 K (c) and 20 K (d), and the inset of (d) shows the OFET structure.**

**Figure S11. Output characteristics after a pre-applied gate voltage of 20 V.**

**Figure S12. Transfer characteristics with and without gate pre-bias.** (a) Transfer characteristics of the OFET device without any pre-applied gate voltage. (b) Transfer characteristics of the OFET device after a pre-applied gate voltage of +40 V.

Applying a pre-polarization bias before measurement significantly changes the conduction behavior. When a +40 V pre-bias is applied, P(VDF-TrFE) is polarized, and the interfacial bound charges effectively modulate the channel carrier distribution, keeping the device in a low conduction state over a wide gate voltage range. The transport layer returns to a conductive state only when the gate voltage becomes sufficiently large to disrupt the polarized state of the ferroelectric layer.

**Figure S13. Transfer Characteristics after Different Gate Pre-Bias Voltages.** (a) Transfer characteristics loop of the OFET device measured after applying a −40 V gate pre-bias. (b) Transfer characteristics loop of the OFET device measured after applying a −20 V gate pre-bias. (c) Transfer characteristics loop of the OFET device measured after applying a 20 V gate pre-bias. (d) Transfer characteristics loop of the OFET device measured after applying a 40 V gate pre-bias.

**Table S1. Field effect mobility (*μ*), on off current ratio (**$\frac{\boldsymbol{I}_{\boldsymbol{on}}}{\boldsymbol{I}_{\boldsymbol{off}}}$**), and threshold voltage (*V_th_*) extracted from transfer measurements under different gate pre-biases.**

| Pre-bias (V) | *μ* (cm^2^·V^−1^·s^−1^) | $\frac{\boldsymbol{I}_{\boldsymbol{on}}}{\boldsymbol{I}_{\boldsymbol{off}}}$ | *V_th_* (V) |
| --- | --- | --- | --- |
| −40 | 2.46 × 10^-4^ (*μ_forward_*)  3.78 × 10^-4^ (μ_reverse_) | 26.02 | 16.83 (*V_th_forward_*)  19.00 (*V _th_reverse_*) |
| −20 | 1.62 × 10^-4^ (*μ_forward_*)  4.20 × 10^-4^ (*μ_reverse_*) | 23.84 | 29.18 (*V _th_forward_*)  19.09 (*V _th_reverse_*) |
| 20 | 106.80 × 10^-4^ (*μ_forward_1_*)  2.64 × 10^-4^ (*μ_forward_2_*)  7.79 × 10^-4^ (*μ_reverse_*) | 37.41 | −12.23 (*V _th_forward_1_*)  14.21 (*V _th_forward_2_*)  14.01 (*V _th_reverse_*) |
| 40 | 101.16 × 10^-4^ (*μ_forward_1_*)  4.94 × 10^-4^ (*μ_forward_2_*)  9.23 × 10^-4^ (*μ_reverse_*) | 56.98 | −12.75 (*V _th_forward_1_*)  4.78 (*V _th_forward_2_*)  15.15 (*V _th_reverse_*) |

It is noted that the mobility values are generally low, likely because the chiral and helical morphology in the channel creates a more tortuous effective transport pathway and increases carrier scattering.


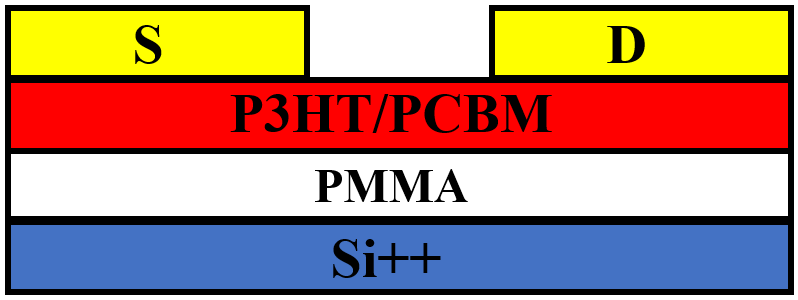


**Figure S14. Output characteristics of the OFET device based on chiral P3HT:PCBM at 300 K, and the inset shows the OFET structure in which the PMMA was used as the dielectric layer.**

No anomalous current peaks are observed in the transfer curves of the PMMA devices . This result indicates that the anomalous peaks are closely associated with the presence of the ferroelectric P(VDF-TrFE) dielectric.

**Figure S15. Output, Transfer, and Gate Current Characteristics of the Bare P(VDF-TrFE) OFET.** (a) Output characteristics of a bare P(VDF-TrFE) film in an OFET structure. (b) Transfer characteristics of a bare P(VDF-TrFE) film in an OFET structure. (c) Gate current recorded during the transfer measurement of the bare P(VDF-TrFE) device.

**Figure S16. Output characteristics measured at different sweep rates, where the voltage step is 0.2 V in (a) and 0.4 V in (b).**

**Figure S17. Output characteristics of the OFET device under 3 mW 532 nm laser illumination (a) and the corresponding gate currents (b). A gate voltage of 20 V was applied prior to the measurements.**

**Figure S18. Output characteristics of the OFET at 200 K.** Output characteristics of the OFET device without (a) and with a 20 V (b), 40 V (c) pre-applied gate voltage at 200 K. With a pre-applied voltage of 40 V, the polarization of the ferroelectric layer strengthens the interface coupling, allowing the transient current to be observed continuously.

**Figure S19. Output characteristics of the OFET at 100 K.** Output characteristics of the OFET device without (a) and with a 20 V (b), 40 V (c) pre-applied gate voltage at 100 K. Lowering the temperature enhances interfacial interactions, making the transient current observable independent of the pre-applied gate voltage.

**Figure S20. Output characteristics of the OFET** **at 100 K under illumination.** Output characteristics of the OFET device at 100 K in the dark (a) and under 1.5 mW 532 nm laser illumination (c), respectively. The corresponding gate currents are shown in (b) and (d). A gate voltage of 40 V was applied prior to the measurements.

**Figure S21. Modulation of *I_SD_*​ by ferroelectric polarization and remanent polarization.** Modulation of *I_SD_*​ by ferroelectric polarization and remanent polarization under dark (a) and illumination (b). The numbers in the figure indicate the magnitude of *V_G_*. It is evident that after P(VDF-TrFE) is polarized, the remanent polarization alone is sufficient to enhance *I_SD_*.

**Figure S22. ESR signals of P3HT:PCBM and P3HT:PCBM/P(VDF-TrFE) heterojunction.** ESR signals at 300 K for P3HT:PCBM (a) and P3HT:PCBM/P(VDF-TrFE) heterojunction with opposite polarization (b and c) under dark and 532 nm laser illumination at different power levels. The polarization direction is shown in the inset of (b) and (c).

**Figure S23. ESR intensity derived from Figure S22.** The distinct light-intensity-dependent behaviors of ESR signals also provide evidence for the presence of interfacial interactions in the device with P3HT:PCBM/P(VDF-TrFE) heterojunction.


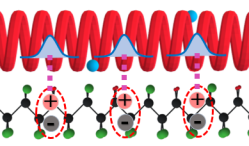


**Figure S24. ESR signals of P3HT:PCBM/P(VDF-TrFE) heterojunction at 100 K.** ESR signals at 100 K for P3HT:PCBM/P(VDF-TrFE) heterojunction under dark and 532 nm laser illumination at different power levels. In contrast to Figure 3b, the P(VDF-TrFE) layer here is polarized in the opposite direction, which is shown in the inset.

**Figure S25. Power dependent CW-ESR spectra of the P3HT:PCBM/P(VDF-TrFE) heterojunction.**

**Figure S26. ESR signals under L- and R-CPL illumination.** A stronger ESR signal is observed under L-CPL, indicating a higher population of unpaired electrons, which in turn leads to an enhanced CMC.

**Figure S27. ESR signals of achiral P3HT:PCBM.** ESR signals of pristine achiral P3HT:PCBM (a) and achiral P3HT:PCBM/P(VDF-TrFE) heterojunction (b) under dark and 532 nm laser illumination.

The ESR results show that illumination enhances the spin related signal in P3HT:PCBM, which is consistent with enhanced charge transfer between achiral P3HT and PCBM. In addition, due to interfacial coupling, the polarization of the P(VDF-TrFE) layer can still modulate the spin polarization in P3HT:PCBM, and the achiral heterostructure also shows a relatively weaker trend in the PCBM electron spin resonance signal compare with the bare P3HT:PCBM


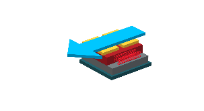

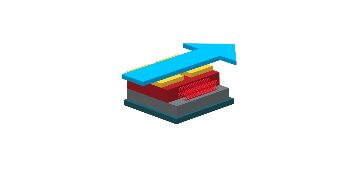

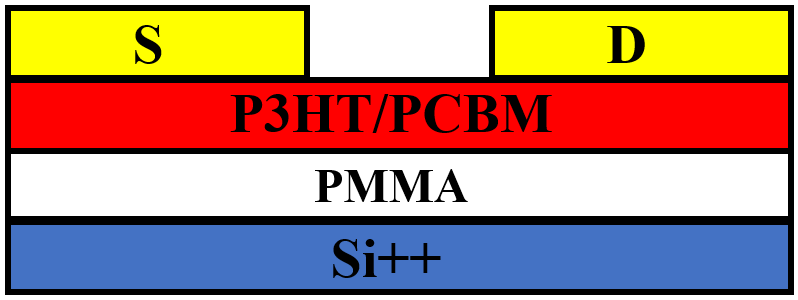


**Figure S28. CMC of the OFET with PMMA as the dielectric layer.** CMC of the OFET under 532 nm laser illumination, and the inset shows the OFET structure in which the PMMA was used as the dielectric layer. In the test, *V_SD_* = −1 V and *V_G_* = −3 V were applied. The insets show the direction of the applied magnetic field (the light blue arrow).

**Figure S29. CMC of achiral OFET.** (a) CMC of OFET devices using achiral P3HT:PCBM as the transport layer under 532 nm LPL illumination. (b) Magnetic-field-dependent *I_SD_* of OFET under 532 nm LPL illumination. The insets show the direction of the applied magnetic field (the light blue arrow) and the polarization states of light.

This demonstrates that although interfacial coupling and spin polarization modulation exist in both types of devices, the necessary condition for generating CMC exists only in chiral P3HT:PCBM. When chirality is absent, B_chiral_ = 0 and B_effective_ = B_external_, so the system is governed only by the external magnetic field and does not produce an additional chirality-related transport. Therefore, chirality is essential for the appearance of CMC.

**Figure S30. CMC of OFET with different P3HT:PCBM mass ratio.** CMC of OFET devices with P3HT:PCBM mass ratios of 2:1 (a) and 1:3 (b) under 532 nm LPL illumination. Magnetic-field-dependent *I_SD_* of OFET devices with P3HT:PCBM mass ratios of 2:1 (c) and 1:3 (d) under 532 nm LPL illumination. The insets show the direction of the applied magnetic field (the light blue arrow).

**Figure S31. Magnetic-field-dependent *I_SD_* of OFET.** Magnetic-field-dependent *I_SD_* of OFET under L-CPL (a), LPL (b) and R-CPL (c) illumination at 300 K. The insets show the direction of the applied magnetic field (the light blue arrow) and the polarization states of light. In all measurements, the gate was pre-biased before testing, and the gate voltage was held at 0 V during data acquisition.


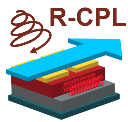

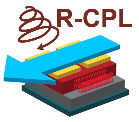


**Figure S32. CMC of the OFET at 300 K under R-CPL illumination.** CMC of the OFET at 300 K under R-CPL illumination, and the insets show the direction of the applied magnetic field (the light blue arrow) and the polarization states of light. In all measurements, the gate was pre-biased before testing, and the gate voltage was held at 0 V during data acquisition.

**Figure S33. Voltage dependence of CMC under LPL (a) and R-CPL (b) illumination at 300 K.** In all measurements, the gate was pre-biased before testing, and the gate voltage was held at 0 V during data acquisition. As shown in the figure, the pre-bias voltage can modulate the CMC by tuning the polarization of P(VDF-TrFE).

**Figure S34. Magnetic-field-dependent *I_SD_* of OFET at 200 K.** Magnetic-field-dependent *I_SD_* of OFET under L-CPL (a), LPL (b) and R-CPL (c) illumination at 200 K. The insets show the direction of the applied magnetic field (the light blue arrow) and the polarization states of light. In all measurements, the gate was pre-biased before testing, and the gate voltage was held at 0 V during data acquisition.

**Figure S35. CMC of the OFET at 200 K**. CMC of the OFET at 200 K under L-CPL (a), LPL (b) and R-CPL (c) illumination, and the insets show the direction of the applied magnetic field (the light blue arrow) and the polarization states of light. In all measurements, the gate was pre-biased before testing, and the gate voltage was held at 0 V during data acquisition.

**Figure S36. Voltage dependence of CMC under L-CPL (a), LPL (b) and R-CPL (c) illumination at 200 K.** In all measurements, the gate was pre-biased before testing, and the gate voltage was held at 0 V during data acquisition.

As the temperature decreases, the applied voltage becomes insufficient to polarize P(VDF-TrFE), thereby weakening the voltage-induced modulation of the CMC. The figure also shows that the CMC variation under ±20 V is not symmetric. This is due to the asymmetric ferroelectric loop caused by the P3HT:PCBM layer (Figure S3), where a +20 V bias is sufficient to polarize P(VDF-TrFE) to some extent, while a –20 V bias is not. In addition, the enhanced interfacial interaction at lower temperatures explains why the polarization-induced CMC change at 200 K is much greater than that at 300 K.

**Figure S37. Magnetic-field-dependent *I_SD_* of OFET at 100 K.** Magnetic-field-dependent *I_SD_* of OFET under L-CPL (a), LPL (b) and R-CPL (c) illumination at 100 K. The insets show the direction of the applied magnetic field (the light blue arrow) and the polarization states of light. In all measurements, the gate was pre-biased before testing, and the gate voltage was held at 0 V during data acquisition.

**Figure S38. CMC of the OFET at 100 K.** CMC of the OFET at 100 K under LPL (a) and R-CPL (b) illumination, and the insets show the direction of the applied magnetic field (the light blue arrow) and the polarization states of light. In all measurements, the gate was pre-biased before testing, and the gate voltage was held at 0 V during data acquisition.

LPL

**Figure S39. Voltage dependence of CMC under LPL illumination at 200 K.** In all measurements, the gate was pre-biased before testing, and the gate voltage was held at 0 V during data acquisition.
